# Device for source position stabilization and beam parameter monitoring at inverse Compton X-ray sources

**DOI:** 10.1107/S1600577519006453

**Published:** 2019-08-07

**Authors:** Benedikt Günther, Martin Dierolf, Klaus Achterhold, Franz Pfeiffer

**Affiliations:** a Department of Physics, Technical University of Munich, James-Franck-Straße 1, 85748 Garching, Germany; b Munich School of BioEngineering, Technical University of Munich, Boltzmannstraße 11, 85748 Garching, Germany; c Department of Diagnostic and Interventional Radiology, Klinikum rechts der Isar, Technical University of Munich, Ismaninger Straße 22, 81675 München, Germany

**Keywords:** inverse Compton X-ray sources, beam-position monitor, active source position stabilization

## Abstract

Compact X-ray sources based on inverse Compton scattering provide brilliant and partially coherent X-rays in a laboratory environment. The cross section for inverse Compton scattering is very small, requiring high-power laser systems as well as small laser and electron beam sizes at the interaction point to generate sufficient flux. Therefore, these systems are very sensitive to distortions which change the overlap between the two beams. In order to monitor X-ray source position, size and flux in parallel to experiments, the beam-position monitor proposed here comprises a small knife edge whose image is acquired with an X-ray camera specifically designed to intercept only a very small fraction of the X-ray beam. Based on the source position drift recorded with the monitor, a closed-loop feedback stabilizes the X-ray source position by adjusting the laser beam trajectory. A decrease of long-term source position drifts by more than one order of magnitude is demonstrated with this device. Consequently, such a closed-loop feedback system which enables stabilization of source position drifts and flux of inverse Compton sources in parallel to experiments has a significant impact on the performance of these sources.

## Abstract 2

An X-ray beam monitor operating in parallel to experiments and adapted to the moderate flux density as well as rather large beam diameter of inverse Compton sources is presented. In conjunction with this device a closed-loop feedback system was developed counteracting the recorded source position drifts and thereby significantly improving the X-ray source position stability.

## 1. Compact X-ray sources based on inverse Compton scattering   

High-brilliance X-rays generated in synchrotron facilities are necessary for a wide range of advanced methods in imaging, *e.g.* phase contrast imaging (Bonse & Hart, 1965 ▸; Snigirev *et al.*, 1995 ▸; Cloetens *et al.*, 1996 ▸; Momose *et al.*, 2003 ▸; Weitkamp *et al.*, 2005 ▸), real-time or high-speed imaging (Berg *et al.*, 2013 ▸; Maire *et al.*, 2016 ▸; Olbinado *et al.*, 2017 ▸) as well as high-resolution imaging (Sakdinawat & Attwood, 2010 ▸), spectroscopy, *e.g.* extended X-ray absorption fine structure (Lee *et al.*, 1981 ▸), or X-ray polarimetry, *e.g.* circular magnetic dichroism (Schütz *et al.*, 1987 ▸; Chen *et al.*, 1995 ▸). Although these powerful techniques can often provide valuable information for biomedical research as well as industrial product screening, their application outside large research centers has been very limited due to the fact that synchrotron facilities are not readily available because of their large dimension and high cost of construction and operation.

Compact X-ray sources based on inverse Compton scattering recently demonstrated sufficient flux and brilliance to potentially transfer many of these techniques into a laboratory environment, (pre-)clinical setting or assembly line. Propagation-based (Ikeura-Sekiguchi *et al.*, 2008 ▸; Gradl *et al.*, 2017 ▸) and grating-based phase contrast imaging (Bech *et al.*, 2009 ▸; Schleede, Meinel *et al.*, 2012 ▸; Schleede, Bech *et al.*, 2012 ▸; Eggl *et al.*, 2015 ▸; Eggl, Schleede *et al.*, 2016 ▸) as well as protein crystallography (Abendroth *et al.*, 2010 ▸) have already been demonstrated at a compact light source which is based on a small electron storage ring. Currently many projects to develop and build inverse Compton sources are ongoing, *e.g.* the Munich Compact Light Source (MuCLS) (Eggl, Dierolf *et al.*, 2016 ▸), the cERL-based laser Compton X-ray source at KEK (Akagi *et al.*, 2016 ▸), STAR (Bacci *et al.*, 2014 ▸, 2016 ▸), Tsinghua Thomson Scattering X-ray Source (Du *et al.*, 2013 ▸), ASU Compact XFEL (Graves *et al.*, 2017 ▸), LLNL Laser Compton X-ray Source (Hwang *et al.*, 2016 ▸), Smart*Light (Luiten, 2016 ▸), NESTOR (Shcherbakov *et al.*, 2013 ▸), SPARC_LAB Thomson Source (Vaccarezza *et al.*, 2016 ▸), ThomX (Variola *et al.*, 2014 ▸). Some of them aim for source sizes below 5 µm which on the one hand should provide sufficient coherence to access the holographic regime, but on the other hand means they require a very stable source position or overlap between electron and laser beam, respectively. In general, temporal thermal gradients influence the radiofrequency phases of the electron accelerator. Additionally, steering of the laser beam can be compromised by thermally induced stress in optics, *e.g.* if those components are placed in close proximity to strong conventional magnets, especially during the magnets’ heat-up. This changes electron beam and/or laser beam position and degrades overlap between these two beams, resulting in a reduced X-ray flux, as well as a shift of the source position. In order to suppress this behavior, we developed a closed-loop X-ray beam-position monitor and stabilization system that is designed to work in parallel to experiments. The system has been implemented at the MuCLS, but can be used in different geometries, too.

The MuCLS, consisting of the Compact Light Source (CLS) developed by Lyncean Technologies Inc. (Fremont, USA) and the beamline including the experimental end-stations developed in-house, is the first commercially available compact synchrotron source based on inverse Compton scattering. As the cross section for inverse Compton scattering is very small (∼6.65 × 10^−29^ m^−2^ for scattering into the full solid angle), high X-ray flux at this source is ensured by a high interaction frequency of 64.91 MHz. To this end, an electron beam revolves in a small storage ring and a laser pulse circulates in a laser ring resonator with a matched frequency, which additionally acts as a passive laser amplifier. This design was proposed by Huang & Ruth (1998 ▸). The MuCLS is constructed to provide X-rays in the range between 15 and 35 keV. Its initial performance after installation at the Munich School of BioEngineering in 2015 was a flux of 1.0 × 10^10^ photons s^−1^ emitted into a cone angle of 4 mrad which is enforced by the exit aperture (Eggl, Dierolf *et al.*, 2016 ▸). A recently installed laser upgrade pushed the flux up to 3 × 10^10^ photons s^−1^ at 35 keV while maintaining small root-mean-square source sizes below 50 µm. This higher flux was reached by significantly increasing the power stored in the laser resonator. As a consequence, the interplay between thermal fluctuations and laser orbit in the cavity became more pronounced. This resulted in stronger shifts of the X-ray source position leading to a degraded X-ray flux (*cf*. Fig. 4*b*), as discussed above.

## 2. An X-ray beam monitor (XBM) for inverse Compton sources   

### 2.1. Design constraints   

In order to monitor and compensate such shifts of the X-ray source position, especially during experiments, a closed-loop feedback system was developed. The main constraints for the design of such a system arise from the feedback frequency on the order of 1 Hz required to compensate the aforementioned drifts, the X-ray flux density provided at state-of-the-art inverse Compton X-ray sources, like the MuCLS, and the respective beamline geometry. Knowing the geometry, two beam-position monitors would be necessary to calculate the source position through extrapolation of the beam trajectory. The one closest to the X-ray source would have to be placed directly downstream of the X-ray exit window of the vacuum chamber, because it is not possible to install any monitor inside the vacuum vessel without severely degrading the laser pulses circulating in the enhancement cavity. This restricts the shortest distance to 1.4 m, where the X-ray beam size is already ∼6 mm in diameter. The second beam-position monitor could be placed only outside the radiation shielding enclosure of the MuCLS just in front of the first experimental setup at a distance of ∼3.0 m. At this distance, the X-ray diameter is 12 mm, which, on the one hand, is too large for commercially available synchrotron beam-position monitor systems; on the other hand, the typical X-ray flux density of at most 2.7 × 10^8^ photons s^−1^ mm^−2^ is orders of magnitude lower than the ones available at synchrotrons, *e.g.* >10^12^ photons s^−1^ mm^−2^ at P05 at Petra III at DESY (DESY, 2018 ▸) or beamline X25 at the NSLS (Muller *et al.*, 2012 ▸). Therefore, common solutions using the X-ray fluorescence of thin diamond screens or polycrystalline quadrant detectors do not deliver sufficient signal levels for online beam-position measurements at flux levels available at inverse Compton sources (Bloomer *et al.*, 2016 ▸). Single-crystalline diamond screens offer sufficient signal for beam-position measurement, as they do not suffer from charge trapping in grain boundaries which reduces detector efficiency (Tromson *et al.*, 2000 ▸). Currently, their size is limited to a diameter of a few millimetres (Bloomer *et al.*, 2016 ▸; Muller *et al.*, 2012 ▸; Morse *et al.*, 2010 ▸). This is smaller than the minimum accessible X-ray beam diameter at the MuCLS of about 6 mm discussed above. Consequently these systems cannot be employed as beam-position monitors for large beam diameters like the ones provided at the MuCLS. Longitudinal segmented ion chambers or split ionization chambers provide information on X-ray flux as well as X-ray beam position, but, like the diamond beam-position monitors, two of these monitors are necessary to determine the X-ray source position and they lack information on the source size of the X-ray beam as well (Menk *et al.*, 2007 ▸). The latter is the most important parameter for ensuring the correct relative timing between the electron and the laser pulse so that inverse Compton scattering takes place at their common focal position.

### 2.2. XBM design   

Considering these constraints, our goal was to develop a more efficient system able to cope with this lower flux density. This can be achieved by recording a geometrically magnified image of a knife edge with a photon-counting hybrid pixel array detector (Eggl, Dierolf *et al.*, 2016 ▸): the extended source leads to a (penumbral) blurring of the knife edge in the recorded image. By fitting an error function to the respective edges, the horizontal and vertical source sizes and the source position can be extracted, if the imaging geometry and detector point-spread function (PSF) are known. The flux of the source can be calculated from the number of photons absorbed in the detector.

While this scheme is very robust and efficient, the implementation used by Eggl, Dierolf *et al.* (2016 ▸) was neither compact nor suited to work in parallel to experiments. Our new setup employing the same principle therefore replaces the previously rather big knife edge, which blocked almost 75% of the X-ray beam, with a tiny version that is inserted only at the very bottom of the beam. The second component of the new implementation is a detector that intercepts only that very small part of the X-ray beam and is placed at a distance of 2.98 m from the interaction point.

**Figure 1:**
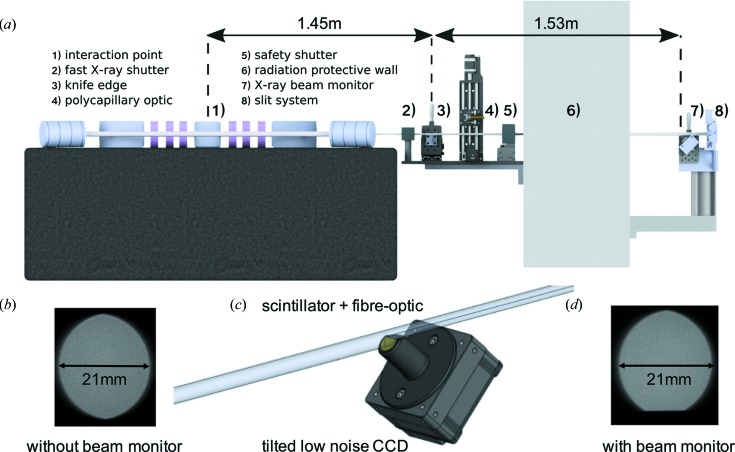
(*a*) Schematic of the MuCLS front-end. The geometry constrains the minimal distance between the X-ray source and knife edge to 1.45 m and the farthest distance for the XBM to 2.98 m in order to be able to stabilize the source position in parallel to experiments. (*b*) The unobstructed X-ray beam. (*c*) Technical drawing of the customized X-ray camera system which is minimally intercepting the X-ray beam at the bottom. (*d*) The resulting X-ray beam usable for experiments when the XBM is inserted.

This way, online X-ray source monitoring becomes possible in parallel to and without negatively affecting experiments. A schematic of the resulting setup is depicted in Fig. 1 ▸(*a*).

As the tungsten knife edge intercepting the X-ray beam from the bottom is located at a distance of 1.45 m from the source point, there is no geometrical magnification in this case. Thus, the X-ray camera has to provide a half-period resolution of at least 10 µm as the X-ray source size is about 50 µm. We chose a commercial Basler Ava 1600-50gm CCD camera (Basler AG, Ahrensburg, Germany) for its low dark noise and small pixel size of 5.5 µm in a very compact housing. A good compromise between high spatial resolution and sufficient X-ray conversion for online measurements in the energy range between 15 and 35 keV is a 10 µm-thick LuAG:Ce single-crystal scintillator. It is covered with a thin reflective aluminium coating in order to enhance light collection on the one hand and inhibit visible light transmission from the environment into the detector on the other hand. Two fiber-optic plates consisting of fibers of 3 µm core diameter guide the visible light photons from the scintillator to the CCD chip. Manufacturing constraints required a thin second fiber-optic plate glued to the CCD chip, while the long fiber-optic plate, to which the scintillator is coupled, is interchangeable. This optic guarantees very efficient transmission while maintaining the resolution of the CCD camera. The long fiber-optic plate of 25 mm length is cut at an angle of 45° at the top in order to allow the rest of the X-ray beam to pass above the housing of the camera when the scintillator is adjusted to intercept only a very small part at the beam’s bottom, *cf*. Fig. 1 ▸(*c*). As a result, the detector system, depicted in Fig. 1 ▸(*a*) at position 7, is tilted by 45° with respect to the optical axis, resulting in an effective vertical pixel size of 3.9 µm. A comparison of Fig. 1 ▸(*b*) displaying the unobstructed X-ray beam with Fig. 1 ▸(*d*), where the XBM is placed into its operating position, demonstrates the feasibility of our approach. Both images are taken with our Andor Zyla 5.5 sCMOS camera (Andor, Belfast, UK) equipped with a 2:1 fiber-optic taper and a 20 µm Gadox scintillator, which is part of our experimental end-station. Customization of the camera according to our design was performed by Crytur (Turnov, Czech Republic). The frames are directly read out from the camera via the EtherLink interface using the *pypylon* library, the official Python wrapper around the *pylon 5* library of Basler. Gain map correction and fits are performed using standard *SciPy* functions (Jones *et al.*, 2001 ▸). The calculated source parameters are sent to the *EPICS* control system of the CLS and used in a software feedback loop to adjust the laser orbit in the cavity.

### 2.3. Characterization of the XBM   

**Figure 2:**
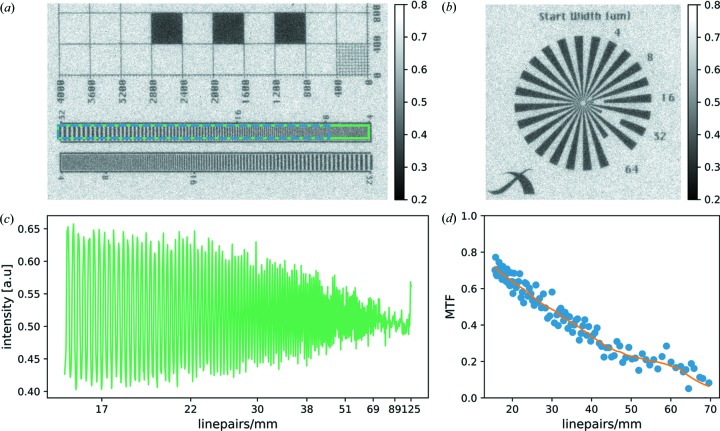
Analysis of the resolution of the XBM. (*a*) The line pattern recorded for determination of the X-ray camera resolution. The Siemens star (*b*) demonstrates isotropic resolution of the camera. (*c*) The intensity modulation of the line pattern indicated with a green box in (*a*) averaged along the lines. (*d*) The MTF values calculated from the raw data depicted in (*c*). The orange line is a moving average (Savitzky–Golay) of the MTF values. At 65 line-pairs mm^−1^ the MTF is 0.1 which fulfills the requirement of resolving the blur caused by a 50 µm X-ray source.

The result of our analysis regarding the camera resolution is displayed in Fig. 2 ▸. For this analysis, the camera and the resolution pattern were oriented parallel to each other, *i.e.* both horizontal and vertical effective pixel sizes are 5.5 µm.

Fig. 2 ▸(*a*) shows a line pattern (Xradia, Pleasanton, USA), which was used for analysis of the modulation transfer function (MTF). The Siemens star in Fig. 2 ▸(*b*) demonstrates isotropic resolution of the camera system. Therefore, MTF analysis of the line pattern enclosed by the green box is representative of the whole camera. Fig. 2 ▸(*c*) depicts the intensity modulation of the line pattern averaged in the vertical direction, *i.e.* along the direction of the lines. The MTF was calculated for the region marked by a blue box in Fig. 2 ▸(*a*) and is plotted in Fig. 2 ▸(*d*). In order to account for substrate absorption, the reference contrast amplitude has been determined as the contrast between the large square structures and the background assuming a constant structure height as well as substrate thickness. The orange line is a moving average (Savitzky–Golay) of the MTF. 65 line-pairs mm^−1^ are resolved at a MTF of 0.1. This corresponds to a resolution limit of ∼8 µm lines which fulfills the 10 µm requirement originally specified for the camera system. Consequently, online monitoring at a frequency of 1 Hz is possible.

**Figure 3:**
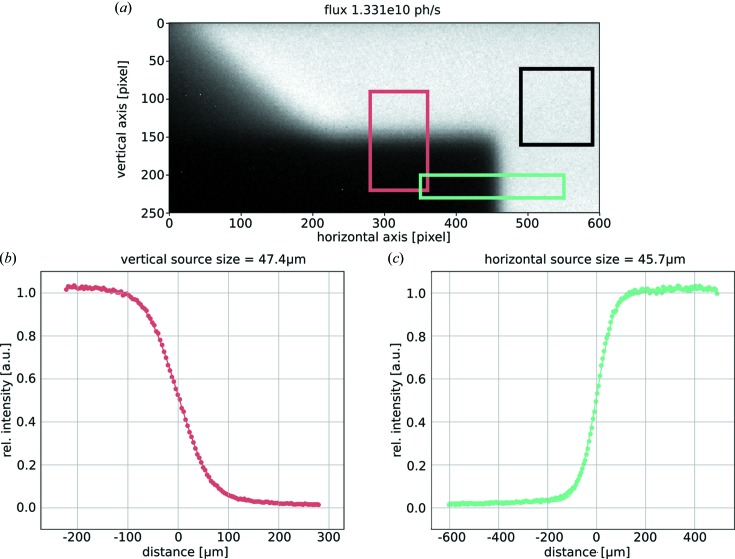
Online operation of the X-ray beam monitoring system. (*a*) The image of the small knife edge inserted into the X-ray beam recorded with the XBM during online X-ray beam stabilization. The individual pixel response of the camera is corrected for with a gain map. The black box indicates the region of interest for X-ray flux determination while the green and red boxes indicate the regions of interest for the error function fit along the vertical and horizontal knife edges. Panels (*b*) and (*c*) depict the measured data as circles and fits as solid lines with the color corresponding to the respective regions of interest.

A typical image is depicted in Fig. 3 ▸(*a*) where the individual pixel response is accounted for with a gain map. The total X-ray flux is determined from the average value of counts within the region of interest shown by the black box with an empirical flux conversion factor. The red and green boxes indicate the regions of interest for the error function fits, which are depicted in Figs. 3 ▸(*b*)–3 ▸(*c*) in the corresponding colors. The raw data are averaged along the direction of the edge before the error function fit. In contrast to photon-counting detectors which exhibit a box-like PSF, any optic in the camera system – in our case a scintillator and a fiber-optic plate – increases the PSF. We determined both the PSF and the flux conversion factor by calibrating the source sizes and the flux measured with the integrating CCD detector to the ones obtained with a photon-counting hybrid pixel array detector (Pilatus 200k, Detris AG, Baden, Switzerland). Its photon counts can be converted into absolute photon flux of the source incorporating knowledge about the spectrum and spectral efficiency of the silicon sensor as well as the setup geometry (Eggl, Dierolf *et al.*, 2016 ▸). Both measurements were recorded in parallel immediately demonstrating the desired capability of the XBM to work in parallel to experiments. Assuming a Gaussian shape of the PSF, a PSF of 5.73 pixels has been calculated in the vertical direction and a PSF of 6.56 pixels in the horizontal one. Source sizes are determined from the measured and PFS-deconvoluted error function fits by multiplying the resulting pixel number with the corresponding effective pixel sizes for the vertical and horizontal direction. The source position can be tracked via the position of the edge in the image.

## 3. Closed-loop X-ray source stabilization   

### 3.1. Correction of source position drift   

First, the offset from the optimum overlap position of laser and electron beam is calculated by comparing the positions recorded with the XBM with the reference value after X-ray tuning. The shift of the source position is then corrected for by adjusting the steering of the laser beam with a motorized mirror mount. In the special case of the MuCLS, we use one of the mirrors of the enhancement cavity to perform this move. This procedure is not limited to correction of laser-driven source position movements in enhancement cavities, but is compatible with single-shot laser or laser recirculator-based inverse Compton sources. The calculated deviation of the source position from the desired one is multiplied with an empirical gain factor which translates the distance into an angular tip/tilt correction for the respective piezoelectric actuators attached to the motorized mirror. As a result, the position of the laser beam is slightly shifted back to its original position correcting the drift of the source position. The lower action limit of the actuator is set to 0.001 V in the software feedback and the determined conversion factor relating the voltage and the source position shift is 0.02 V µm^−1^. Therefore, the minimum motion of the source position by the feedback is 0.05 µm. This is smaller than the sub-pixel accuracy of the source position determination of ±0.3–0.4 µm given as the standard deviation of the fit parameters of the error function fits to the respective edges. Consequently, the sensitivity of the system is limited by the determination of the source position. The feedback usually runs with an update frequency of 1 Hz, which is more than sufficient to correct the substantially slower thermal drifts and allows one to operate the XBM with an exposure time of 0.5 s. Image readout and calculation of the source parameters take another ∼0.07 s, online visualization of the acquired data and the knife-edge fits inside the analysis loop takes ∼0.25 s (but could simply be turned off). The design frequency of 1 Hz is achieved by implementing an additional wait time. In particular, the beam-position monitor is also used for the initial optimization of the overlap between the laser beam and electron beam. Also, in this case, the acquisition time of 0.5 s is sufficient to still obtain reliable results at a flux of around 10^9^ photons s^−1^ which is one order of magnitude lower than the typical case. Consequently, the acquisition time could be significantly reduced for the purpose of beam stabilization, but ultimately the feedback frequency is limited by the flux density provided by the X-ray source at the position of the beam monitor.

### 3.2. Evaluation of the XBM and its performance in beam stabilization   

**Figure 4:**
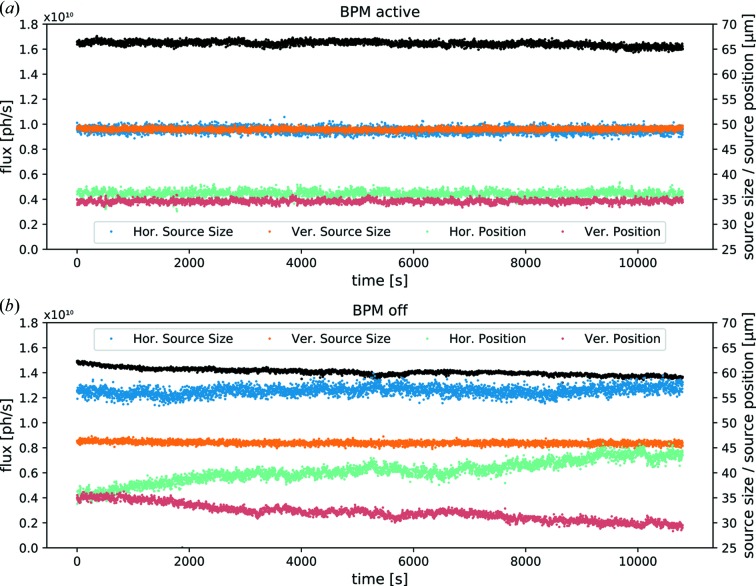
Comparison of the performance of the MuCLS with active source stabilization (*a*) and without stabilization (*b*) over the course of 3 h. Source sizes (blue and orange) are relatively stable in both cases. In contrast to this behavior, the source positions (green and red) drift significantly if no active stabilization is performed, while they remain perfectly stable if the closed-loop feedback system is running. Actively pinning the source position improves X-ray flux stability in addition, as the optimum overlap between laser and electron beam is maintained. The source positions are shifted with an artificial offset of +35 µm for clarity. Quantitative values are shown in Table 1 ▸.

The benefit of the feedback on source position is clearly visible by comparing the two 3 h runs displayed in Fig. 4 ▸ where the closed-loop feedback was active in (*a*) and turned off in (*b*). This duration was chosen because it reflects typical timescales for experiments frequently performed at the MuCLS, *e.g.* grating-based phase contrast tomography (Eggl *et al.*, 2015 ▸). Quantitative numbers of the MuCLS performance during these runs are given in Table 1 ▸. A general observation is that variances of source position, source size as well as flux are substantially lower, if the feedback is running.

However, even more important for experiments are the maximum drifts. A drop in the X-ray flux of 8% is observed over the course of 3 h when the feedback is turned off compared with only 2% when the beam stabilization is active. Instead of position drifts of 8.94 µm over the period of 3 h in the horizontal direction and −5.75 µm in the vertical direction, the X-ray source position remains almost perfectly stable when the feedback is active, resulting in drifts of 0.6 µm in the horizontal direction and −0.46 µm in the vertical one. Absolute shifts well below 1 µm over the course of 3 h correspond to a relative shift of ∼1% of the source size when the feedback is running compared with shifts of up to ∼16% when the feedback is turned off. The observed improvement amounts to a factor of 14.9 in the horizontal source position stability and 12.5 in the vertical one, which is a reduction by more than one order of magnitude.

## 4. Significance of X-ray source position stabilization   

Drifts of the source position presently limit the applicability of inverse Compton sources to certain types of experiments, *e.g.* scanning microscopy or phase contrast imaging. It has been demonstrated here that a beam-position monitor consisting of a knife edge and a customized commercial CCD camera is sufficient to determine X-ray source parameters, such as X-ray flux, source size and position online. It therefore overcomes the limited applicability of commercial synchrotron beam-position monitors at this class of sources due to the moderate flux densities and large beam diameters. In addition, it allows one to stabilize the X-ray source in combination with a feedback system. This is achieved by acting upon changes in X-ray source position and adjusting the steering of the laser pulse within the resonator such that the shift of the source position is compensated. Most importantly, this is possible in parallel to and without impairing experiments. Instead of drifts of the source position of up to 9 µm within 3 h, the feedback reduces the amplitude of the drift by more than one order of magnitude.

This work enables very position-sensitive experiments to be carried out at compact inverse Compton X-ray sources in the future. Moreover, this X-ray beam stabilization scheme is not limited to inverse Compton sources. Any kind of X-ray source providing a sufficient flux density and incorporating elements which can be used as actuators to actively influence the source position, like electron beam optics and/or motorized laser beam optics, can exploit our proposed scheme for source position stabilization. Instead of adjusting a mirror of the laser beam to keep the source position constant, an electron optic would have to be adjusted to steer the electron beam back to its original position.

## Figures and Tables

**Table 1:** Effect of the XBM on source stability The origin of the source position is defined at the optimum overlap between laser and electron beam. Relative motion of the source position has physical consequences as it has a detrimental effect on source position sensitive X-ray experiments. Std: standard deviation.

	Active				Off			
XBM feedback	Mean	Variance	Std	3 h drift	Mean	Variance	Std	3 h drift
Horizontal position (µm)	1.08	0.36	0.60	0.67	5.31	4.79	2.19	8.94
Vertical position (µm)	−0.40	0.13	0.35	−0.46	2.94	2.70	1.64	−5.75
Horizontal size (µm)	48.80	0.32	0.57	−0.61	56.38	0.76	0.87	1.08
Vertical size (µm)	48.99	0.09	0.29	0.36	45.97	0.12	0.35	−0.71
Flux (10^10^ photons s^−1^)	1.64	0.0003	0.02	−0.03	1.41	0.001	0.03	−0.12
